# Liver stiffness for predicting adverse cardiac events in chinese patients with heart failure: a two-year prospective study

**DOI:** 10.1186/s12872-022-02497-w

**Published:** 2022-02-14

**Authors:** Qian Wang, Yuqing Song, Qiming Wu, Qian Dong, Song Yang

**Affiliations:** 1 Department of Cardiovascular Diseases, Beijing Ditan Hospital of Capital Medical University, Beijing, 100015 China; 2 Center of Hepatology, Beijing Ditan Hospital of Capital Medical University, Beijing, 100015 China; 3 Center of Hepatology, Beijing Ditan Teaching Hospital, Peking University Health Science Center, Beijing, 100015 China; 4 Department of Hepatology, The Fourth People’s Hospital of Qinghai Province, Xining, 81000 China

**Keywords:** Heart failure, Liver stiffness, Prognosis, Tricuspid annual plane systolic excursion, Echocardiography

## Abstract

**Background:**

To investigate whether liver stiffness (LS) can predict adverse cardiac events in Chinese patients with heart failure (HF).

**Methods:**

A total of 53 hospitalized patients with HF were enrolled, and LS and tricuspid annual plane systolic excursion (TAPSE) were determined with Fibroscan® and echocardiography before discharge. They were divided into two groups: high LS group (LS > 6.9 Kpa, n = 23) and low LS group (LS ≤ 6.9 Kpa, n = 30). Patients were followed up for 24 months at an interval of 3 months. The endpoint of follow-up was death or rehospitalization for HF.

**Results:**

All patients were followed up for 24 months or until the endpoint. Patients in the high LS group had lower platelet count (*P* = 0.014), lower creatine clear rate (*P* = 0.014), higher level of B-type natriuretic peptide at discharge (*P* = 0.012), and lower TAPSE (*P* < 0.001) than those in the low LS group. During 24 months of follow-up, 3 (5.7%) deaths and 21 (39.6%) hospitalizations for HF were observed. Patients in the high LS group had a higher rate of death/rehospitalization than those in the low LS group (Hazard ratio 4.81; 95% confidence interval 1.69–13.7, *P* = 0.003) after adjustment for age, sex, platelet count, creatine clear rate, and B-type natriuretic peptide level. Moreover, TAPSE ≤ 16 could predict adverse cardiac events with an HR of 6.63 (95% confidence interval 1.69–13.7, *P* = 0.004) after adjustment for age, sex, platelet count, creatine clear rate, and B-type natriuretic peptide level.

**Conclusion:**

LS and TAPSE could be used to predict worse outcomes in patients with HF.

## Background

Patients with heart failure (HF) have high rates of mortality and rehospitalization, and multiple studies are trying to identify predictors of worse prognosis in these patients [1, 2]. Recent studies show that right ventricle (RV) dysfunction plays a key role in the hemodynamics and prognosis of patients with HF. Indeed, RV failure implies an increased risk of cardiac adverse events, regardless of the left ventricle dysfunction degree [3, 4]. In patients with RV failure, increased right-sided filling pressure may cause abnormal liver function and liver congestion. Notably, liver congestion may cause an increase in liver stiffness (LS), which can be quantified by LS measurements [5].

LS measurements with transient elastography (FibroScan®) were first developed to evaluate liver fibrosis noninvasively. Further studies show that decompensated HF may increase LS value measured by transient elastography [6], and LS may reflect right-side filling pressure in patients with HF [7]. Additionally, several recent studies demonstrate that LS measured by transient elastography is promising for prognosis prediction in patients with HF [8]. However, data about the efficacy of LS in predicting the long-term prognosis of HF patients are still limited. Moreover, tricuspid annular plane systolic excursion (TAPSE) is reported to be related to the prognosis of patients with HF [9]. However, data about the long-term follow-up of TAPSE and prognosis of Chinese patients are limited. Accordingly, in this study, we prospectively enrolled patients with HF at a tertiary hospital to evaluate the efficacy of LS and TAPSE in predicting 2-year adverse events.

## Methods

### Patients

Hospitalized patients with HF were screened between June 2018 and December 2018 at Beijing Ditan Hospital of Capital Medical University. HF was diagnosed by experienced attending physicians according to the 2018 Chinese Guidelines for the Diagnosis and Treatment of Chronic Heart Failure [10] based on typical signs and symptoms, corroborated by elevated natriuretic peptide levels and/or objective evidence of cardiogenic pulmonary or systemic congestion from chest X-rays. Particularly, typical symptoms and signs include dyspnea and signs of pulmonary and/or peripheral congestion. The diagnosis was coded based on International Classification of Diseases-10. Patients underwent individualized therapy and were discharged according to related guidelines. Patients were excluded based on the following criteria: ① previous diagnosis of chronic liver disease; ②HBsAg positive and/or HCV RNA positive; ③ultrasound, abdominal CT, and/or MRI showed signs of chronic liver diseases, cirrhosis, portal hypertension, and/or liver cancer; ④ patients who could not get valid LS tests.

### Follow-up and Endpoints

A total of 53 patients were enrolled and followed up every 3 months by clinical visits or through telephone interviews. All patients were followed up for 24 months or till the primary endpoint. The patients were divided into the following two groups: high LS group (LS > 6.9 Kpa, n = 23) and low LS group (LS ≤ 6.9 Kp, n = 30) according to a previous study [7]. The primary endpoint was death or rehospitalization for HF. For patients who experienced more than one cardiovascular events, only the first event was used for analysis.

### Demographic information and laboratory examinations

Data regarding demographic information, physical examination, laboratory index, echocardiography, and co-morbidities medications were documented. Hypertension [11] and diabetes [12] were diagnosed according to related guidelines. All patients underwent tests of complete blood count, liver function, kidney function, and serum B-type natriuretic peptide (BNP) when discharged.

### Echocardiography and LS measurements

Echocardiography was performed with an EPIQ 5 device (Philips, Netherlands) when patients were discharged by an experienced ultrasonography physician. Particularly, TAPSE was measured by M-mode echocardiography with the cursor optimally aligned along the direction of the tricuspid lateral annulus in the apical four-chamber view. Right ventricular dysfunction was defined as TAPSE ≤ 16 mm according to related guidelines [13]. LS measurements were performed with a Fibroscan® device (Echosens, France) according to the manufacturer’s instructions before patients were discharged. The measurements were expressed in Kpa and corresponded to the median values of 10 acquisitions with a success rate of at least 60% and an interquartile range (IQR) of less than 10% [14].

### Statistical analysis

All statistical analyses were performed using the R Statistics version 4.1.1 (Vienna, Austria) and SPSS 22. 0 (Chicago, IL, USA). All data were tested for normal distribution and homogeneity of variance. Continuous variables are expressed as mean values ± standard deviation (mean ± SD) or median (interquartile interval) [M (Q25-Q75)], and categorical variables are expressed as percentages (%). T-test was used to compare normal distribution measurement data between the two groups. Spearman correlation analysis was used to show the correlation between different parameters. Wilcoxon rank-sum test and Mann–Whitney test were used for the comparison of non-normal distribution continuous variables. The Chi-square test was used for the comparison of categorical variables. Kaplan–Meier curves were constructed to examine the time to an event and were analyzed using a log-rank test. Cox proportional hazards regression models were used to determine the independent association of LS/TAPSE with the risk of adverse outcomes, and age, sex, and potential covariates were included in the model. The performance of the model was evaluated by the concordance index (C-index). All *P*-values reported were two-sided, and *P* < 0.05 was considered statistically significant. Reporting of the study conforms to Strengthening the Reporting of Observational Studies in Epidemiology and the Enhancing the Quality and Transparency of Health Research guidelines.

## Results

### Study population

Patients were divided into two groups: High LS group (LS > 6.9 Kpa, n = 23) and low LS group (LS ≤ 6.9 Kpa, n = 30). Demographic characteristics and clinical data of the total population and two groups are shown in Table 1. There was no difference in age, sex ratio, or ratio of New York Heart Association class III/IV patients in different LS groups. However, patients in the high LS group showed higher BNP level (*P* = 0.012) and lower platelet count (*P* = 0.014), creatinine clearance rate (Ccr; *P* = 0.014), and TAPSE (*P* < 0.001) than those in the low LS group.

**Table 1:** Clinical characteristics of the study population

	Overall (n = 53)	LS ≤ 6.9 kPa (n = 30)	LS > 6.9 kPa (n = 23)	*P* value
*Clinical characteristics*				
Age, years	65.5 ± 12.8	64.36 ± 13.56	67.04 ± 11.94	*P* = 0.457
Male, n%	39 (73.6)	22 (73.3)	17 (73.9)	*P* = 0.962
NYHA class III/IV, n%	44 (83.0)	23 (76.7)	21 (91.3)	*P* = 0.300
Systolic BP, mmHg	122.79 ± 17.47	125.63 ± 17.38	119.09 ± 17.25	*P* = 0.178
Diastolic BP, mmHg	71.49 ± 10.13	73.33 ± 10.42	69.09 ± 9.42	*P* = 0.132
*Etiology*				
Ischemic heart disease, n%	36 (67.9)	18 (60.0)	18 (78.3)	*P* = 0.265
Cardiomyopathy, n%	9 (17.0)	7 (23.3)	2 (8.7)	*P* = 0.300
Valvular heart disease, n%	5 (9.4)	2 (6.7)	3 (13.0)	*P* = 0.754
Unknown, n%	3 (5.7)	3 (10.0)	0 (0)	*P* = 0.336
*Comorbidities*				
AF, n%	8 (15.1)	4 (13.3)	4 (17.4)	*P* = 0.983
Hypertension*, n%	39 (73.6)	22 (73.3)	17 (73.9)	*P* = 0.962
Diabetes*, n%	23 (43.4)	11 (36.7)	12 (52.2)	*P* = 0.259
*Laboratory parameters*				
Hemoglobin, g/l	126.5 ± 20.9	128.9 ± 20.2	123.5 ± 21.9	*P* = 0.358
Platelet count,10^9^/L	192 (161.5–249)	212.5 (177.5–266.5)	175 (139–230)	*P* = 0.014
Ccr$, %	73.14 ± 25.78	79.97 ± 23.30	62.46 ± 25.83	*P* = 0.014
Log_2_ (BNP) $, pg/ml	8.38 ± 1.88	7.80 ± 1.74	9.14 ± 1.83	*P* = 0.009
AST, U/L	19.9 (15.8–29.4)	19.3 (15.8–23.6)	20.0 (14.85–30.25)	*P* = 0.560
ALT, U/L	19.6 (13.15–28.5)	18.7 (13.15–27.1)	20.7 (13.1–30.45)	*P* = 0.872
TBIL, μmol/l	12.8 (7.85–17.9)	12.6 (7.2–16.0)	13.6 (8.4–19.9)	*P* = 0.238
DBIL, μmol/l	4.5 (2.9–7.3)	4.3 (2.5–5.5)	5.7 (3.4–8.2)	*P* = 0.065
Albumin, g/L	38.4 (35.9–41.0)	38.6 (35.0–41.2)	38.3 (35.9–40.8)	*P* = 0.970
PT, sec	12.1 (11.5–13.0)	11.9 (11.5–13.0)	12.3 (11.5–14.3)	*P* = 0.267
CHE, U/L	6712.2 ± 2391.9	7255.4 ± 2337.7	6003.6 ± 2322.0	*P* = 0.058
*Echocardiographic parameters*				
LVEDD, mm	58.0 (51.5–62.0)	57.5 (52.0–61.5)	59.0 (48.5–62.0)	*P* = 0.844
LVEF, n%	40.32 ± 11.20	39.95 ± 9.97	40.81 ± 12.8	*P* = 0.785
LADD, mm	44.11 ± 7.26	43.47 ± 5.72	44.96 ± 8.96	*P* = 0.465
Moderate/Severe MR, n%	25 (47.2)	12 (40)	13 (56.5)	*P* = 0.232
Moderate/Severe TR, n%	17 (32.1)	8 (26.7)	9 (39.1)	*P* = 0.335
TAPSE ≤ 16 mm, n%	18 (34.0)	4 (13.3)	14 (60.9)	*P* < 0.001
IVC, mm	19.6 ± 3.6 (n^#^ = 23)	17.5 ± 4.0 (n^#^ = 9)	21.2 ± 2.3 (n^#^ = 14)	*P* = 0.011
TRPG, mmHg	30.0 (22.0–50.0)	26.0 (20.5–41.0)	35.5 (25.5–60.0)	*P* = 0.060
*Medications*				
Beta blockers	51 (96.2)	29 (96.7)	22 (95.7)	*P* = 1.000
ACEIs/ARBs, n%	34 (64.2)	19 (63.3)	15 (65.2)	*P* = 0.887
Sacubitril/valsartan, n%	15 (28.3)	9 (30.0)	6 (26.1)	*P* = 0.754
Diuretics	51 (96.2)	28 (93.3)	23 (100.0)	*P* = 0.472

ACEIs: angiotensin converting enzyme inhibitors; AF: atrial fibrillation; ALT: alanine aminotransferase; ARBs: Angiotensin Receptor Blockers; AST: aspartate aminotransferase; BNP: B-type natriuretic peptide; BP: blood pressure; CAD: coronary artery disease; Ccr: creatinine clearance rate; CHE: cholinesterase; DBIL: direct bilirubin; IVC: inferior vena cava; LADD: left atrium diastole diameter; LS: liver stiffness; LVEDD: left ventricular end-diastolic dimension; LVEF: left ventricular ejection fraction; MR: mitral regurgitation; NYHA: New York Heart Association; PT: Prothrombin Time; TAPSE: tricuspid annual plane systolic excursion; TBIL: total bilirubin; TR: tricuspid regurgitation. TRPG: Tricuspid Regurgitation Pressure Gradient

^#^: data available in partial patients;

^*^: Diagnosis of Hypertension and diabetes is according to related guidelines [11, 12]

^$^: Ccr (ml/min) = (× 0.85 if female) { (140 − age) × body weight (kg)}∕{ 72 × serum creatine (mg/dl)}; log2 (BNP, pg/ml) = 2 based log-transformation of B-type natriuretic peptide (pg/ml)

### LS for prediction of adverse events in HF patients

**Fig. 1:**
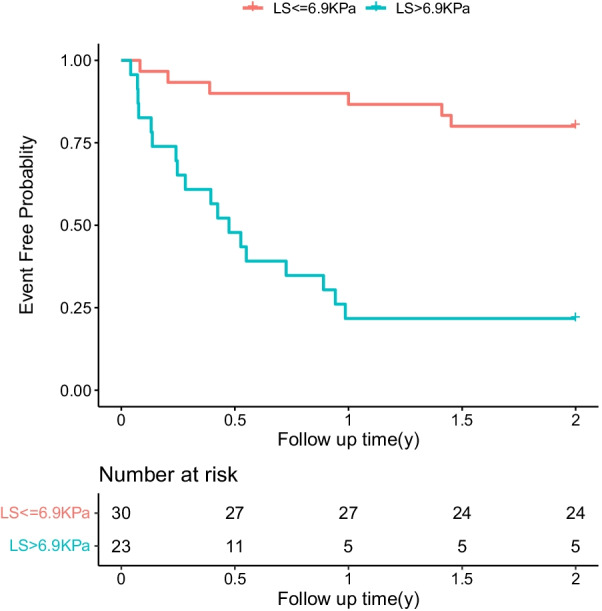
Kaplan–Meier analysis between patients in the high (LS > 6.9 Kpa) and low (LS ≤ 6.9 Kpa) LS groups. The high LS group: n = 23; the low LS group: n = 30. LS: liver stiffness

All patients were followed up for 24 months or till the endpoint. The minimum follow-up time was 15 days, the maximum follow-up time was 730 days, and the median follow-up duration was 730 days (IQR 149–730 days). A total of 24 (45.3%) patients experienced adverse events during the 24-month follow-up, which included three deaths (5.7%). Kaplan–Meier curves showed that patients in the high LS group showed a higher risk of endpoint events than those in the low LS group (log-rank test χ^2^ = 16.648, *p* < 0.001) (Fig. 1). To select the parameters for the multivariate cox proportional hazards model, we first used the univariate Cox proportional hazard model to identify the parameters related to 2-year prognosis in this cohort. We found that LS, Ccr, platelet, BNP, and TAPSE ≤ 16 mm could predict the prognosis of patients in this cohort (all Ps < 0.05). Spearman correlation analysis showed that TAPSE was significantly correlated with LS (correlation coefficient =  − 0.395, *P* < 0.001). We built two multivariate Cox proportional hazard models to show the prediction value of LS and TAPSE.

In univariate Cox regression analysis, the risk of adverse cardiac events in HF patients with LS > 6.9 Kpa increased by 6.86 times (95% confidence interval [CI] 3.08–20.06; *p* < 0.001) compared with that in patients with LS ≤ 6.9 Kpa. Multivariable Cox regression analysis showed that LS > 6.9 Kpa could still predict adverse cardiac events with a hazard ratio (HR) of 4.81 (95% CI 1.69–13.7, *P* = 0.003) after adjusting for age, sex, Ccr, log_2_ (BNP), and platelet count (Table 2). The C-index of this model was 0.798 (95% CI 0.714–0.882).

**Table 2:** Association between LS > 6.9Kpa and risk of cardiac event of patients with HF

	Univariate		Multivariable	
	HR	*P* value	HR	*P* value
Age (years)	1.00 (0.97–1.03)	0.945	1.01 (0.97–1.05)	0.796
Sex (Male/Female)	0.51 (0.17–1.49)	0.218	0.72 (0.27–1.98)	0.528
LS > 6.9 kPa	7.86 (3.08–20.06)	< 0.001	2.96 (1.10–8.02)	0.032
Ccr (per 1 ml/min increase)	0.98 (0.97–1.00)	0.016	1.00 (0.98–1.01)	0.660
PLT (per 1 × 10^9^/L increase)	0.99 (0.99–1.00)	0.036	1.00 (0.99–1.00)	0.703
Log_2_BNP (per 1 increase)	1.35 (1.08–1.70)	0.010	1.26 (0.97–1.63)	0.091

Ccr: creatinine clearance rate; HF: heart failure; LS: liver stiffness; PLT: platelet;

Log_2_ (BNP) = 2 based log-transformation of B-type natriuretic peptide

Multivariable Cox analysis model 1 included LS > 6.9 kPa, age, sex, Ccr, log_2_ (BNP), and platelet

Univariate Cox regression analysis showed that per 1 kPa increase of LS could predict the risk of adverse events with an HR of 1.08 (95% CI 1.04–1.14, *P* = 0.001). We further built a multivariable Cox regression analysis model with LS, age, sex, Ccr, log2 (BNP), and platelet count. As shown in Table 3, in this model, per 1 kPa increase of LS could still predict the risk of adverse events with an HR of 1.10 (95% CI 1.03–1.17, *P* = 0.004). In addition, 1 log2BNP increase could also predict adverse events with an HR of 1.31 (95% CI 1.01–1.70, *P* = 0.041). The C-index of the model was 0.779 (95% CI 0.691–0.867).

**Table 3:** Association between LS value and risk of cardiac event of patients with HF

	Univariate		Multivariable	
	HR	*P* value	HR	*P* value
Age (years)	1.00 (0.97–1.03)	0.945	1.00 (0.96–1.04)	0.874
Sex (Male/Female)	0.51 (0.17–1.49)	0.218	0.63 (0.22–1.75)	0.373
LS (per 1 kPa increase)	1.08 (1.04–1.14)	0.001	1.10 (1.03–1.17)	0.004
Ccr (per 1 ml/min increase)	0.98 (0.97–1.00)	0.016	1.00 (0.97–1.01)	0.210
PLT (per 1 × 10^9^/L increase)	0.99 (0.99–1.00)	0.036	1.00 (0.99–1.01)	0. 566
Log_2_BNP (per 1 increase)	1.35 (1.08–1.70)	0.010	1.31 (1.01–1.70)	0. 041

Ccr: creatinine clearance rate; HF: heart failure; LS: liver stiffness; PLT: platelet;

Log2 (BNP) = 2 based log-transformation of B-type natriuretic peptide;

Multivariable Cox analysis model 2 included LS, age, sex, Ccr, log_2_ (BNP), and platelet

### TAPSE for prediction of adverse events in HF patients

**Fig. 2:**
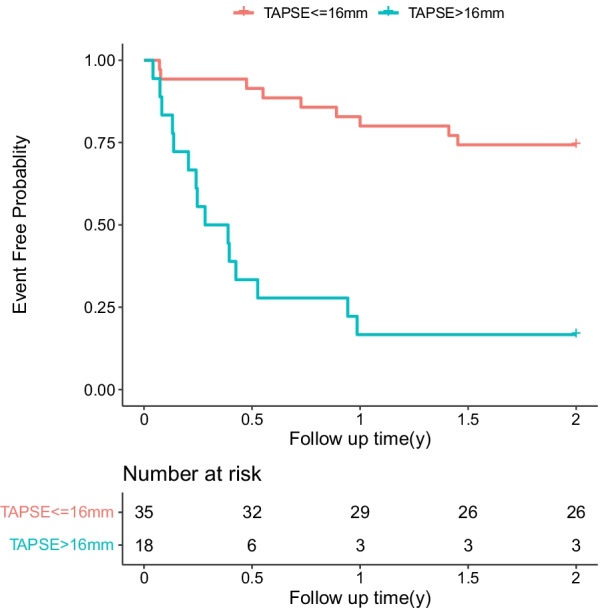
Kaplan–Meier analysis between patients with TAPSE > 16 mm (n = 35) and those with TAPSE ≤ 16 mm (n = 18). TAPSE: tricuspid annual plane systolic excursion

Because TAPSE is a key echocardiography parameter to reflect RV function, Kaplan–Meier curves were constructed to compare the prognosis between HF patients with TAPSE ≤ 16 mm and those with TAPSE > 16 mm (Fig. 2). Patients with TAPSE ≤ 16 mm showed a higher risk of cardiac events than those with TAPSE > 16 mm (log-rank test χ^2^ = 15.840, *P* < 0.001). Univariate Cox regression analysis showed that the risk of adverse cardiac events increased by 5.82 times (95% CI 2.90–16.01; *p* < 0.001) in HF patients with TAPSE ≤ 16 mm compared with that that in HF patients with TAPSE > 16 mm. Multivariable Cox regression analysis showed that TAPSE ≤ 16 could still predict adverse cardiac events with an HR of 6.63 (95% CI 1.84–23.96, *P* = 0.004) after adjusting for age, sex, Ccr, log_2_ (BNP), and platelet count (Table 4). The C-index of this model was 0.801 (95% CI 0.703–0.899).

**Table 4:** Association between TAPSE ≤ 16 mm and risk of cardiac event of patients with HF

	Univariate		Multivariable	
	HR	*P* value	HR	*P* value
Age (years)	1.00 (0.97–1.03)	0.945	1.01 (0.97–1.05)	0.696
Sex (Male/Female)	0.51 (0.17–1.49)	0.218	0.49 (0.17–1.40)	0.182
Ccr (per 1 ml/min increase)	0.98 (0.97–1.00)	0.004	0.99 (0.97–1.01)	0.424
PLT (per 1 × 10^9^/L increase)	0.99 (0.99–1.00)	0.036	1.00 (0.99–1.01)	0.549
Log_2_BNP (per 1 increase)	1.35 (1.08–1.70)	0.010	1.15 (0.85–1.55)	0.376
TAPSE ≤ 16 mm	6.82 (2.90–16.01)	< 0.001	3.31 (1.09–10.07)	0.035

Ccr: creatinine clearance rate; HF: heart failure; LS: liver stiffness; PLT: platelet; TAPSE: tricuspid annual plane systolic excursion;

Log2 (BNP) = 2 based log-transformation of B-type natriuretic peptide

Multivariable Cox analysis model 3 included TAPSE ≤ 16 mm, age, sex, Ccr, log_2_ (BNP),and platelet

## Discussion

Recent studies have shown that LS could be potentially used to predict the prognosis of patients with HF. However, the cut-off of LS for predicting prognosis is controversial, and long-term follow-up data of patients are lacking [15]. In Saito et al.’s study [16], 105 acute decompensated HF patients were followed up for an average of 153 days, and HF patients with LS > 8.8 Kpa had a significantly higher rate of cardiac events. However, in Taniguchi et al.’s study [7], 171 HF patients were followed up for an average of 203 days, and patients with LS > 6.9 Kpa had a higher risk of cardiac events. In this present study, we chose 6.9 Kpa as the cut-off of LS and showed that LS > 6.9 Kpa could predict a higher risk of cardiac events in 24 months of follow-up. The present findings support the use of LS in the long-term management of patients with HF.

The rationale of using LS to predict prognosis of HF is that LS is correlated with central venous pressure, a comprehensive index to reflect both RV dysfunction and preload increase related to left ventricular dysfunction [17, 18]. Besides LS measured by Fibroscan, Fibrosis-4 and non-alcoholic fatty liver disease fibrosis scores have also been used to predict the prognosis of HF patients [19, 20]. Moreover, recently, Saito et al. found that spleen stiffness measured by two-dimensional shear-wave elastography could be used to predict the prognosis of patients with HF [21]. In addition, abdominal viscera stiffness is also promising for predicting the prognosis of HF patients.

As LS measurement is not always available for HF patients, TAPSE based on echocardiography measurement is another well-established parameter to evaluate RV function. In this study, HF patients with TAPSE ≤ 16 mm had a higher risk of cardiac events in 2 years of follow-up. This result is in accordance with that in previous studies [22, 23]. These findings suggest that TAPSE can also be an important parameter for predicting the prognosis of patients with HF.

This study has several limitations. First, this was a single-centered study, and the sample size was small. Further multi-centered studies with a large sample size are needed to verify the present findings in Chinese HF patients. Second, the dynamics of LS during the follow-up could be monitored to determine its correlation with patients’ prognosis.

## Conclusions

This study showed that LS and TAPSE could be used for predicting the 2-year prognosis of patients with HF. The present findings suggest that HF patients with LS > 6.9 Kpa and/or TAPSE ≤ 16 mm should be monitored more closely for possible adverse cardiac events.

## Data Availability

The datasets generated and/or analyzed during the current study are not publicly available due to limitations of ethical approval involving the patient data and anonymity but are available from the corresponding author on reasonable request.

## Abbreviations

BNP: B-type natriuretic peptide
Ccr: Creatinine clearance
CI: Confidence interval
HF: Heart failure
IQR: Interquartile range
HR: Hazard ratio
LS: Liver stiffness
RV: Right ventricle
TAPSE: Tricuspid annual plane systolic excursion
